# Highly improved convergence approach incorporating edge conditions for scattering analysis of graphene gratings

**DOI:** 10.1038/s41598-020-69827-w

**Published:** 2020-07-30

**Authors:** Ruey-Bing Hwang

**Affiliations:** 1 Institute of Communications Engineering, College of Electrical and Computer Engineering, National Chiao Tung University, Hsinchu, 30050 Taiwan; 2 Center for mmWave Smart Radar Systems and Technologies, National Chiao Tung University, Hsinchu, 30050 Taiwan

**Keywords:** Optical properties and devices, Computational science

## Abstract

This research developed an effective and efficient approach for improving the slow convergence in the scattering analysis of a one-dimensional graphene grating, made of a periodic array of parallel graphene strips, illuminated by a TM-polarized plane wave. Specifically, the electric fields over the graphene strips and slit regions in a unit cell are individually expressed as an expansion of local basis functions inherently satisfying edge conditions. Interestingly, convergence rate is highly improved compared to the customary and modified Fourier modal method. Additionally, with the aid of local basis functions, the Gibbs phenomenon occurring at both edges of graphene strip can be removed.

## Introduction

The plane wave scattering by a one-dimensional (1D) grating made up of dielectric or metallic mediums has been intensively and extensively studied. Some numerical methods such as the rigorous coupled-wave analysis (RCWA)^1^, the modal theory for dielectric and finitely conducting gratings^2^, and the modal transmission-line method^3–5^ were developed to accurately calculate scattering characteristics of gratings. Moreover, graphene-based grating composed of graphene sheet has been a continued research interest^6–14^ in both theoretical studies and practical applications in recent years.

In the RCWA method, both the permittivity function of a periodic medium and electromagnetic fields are expanded into Fourier series and Floquet-Fourier series, respectively. Therefore, the electromagnetic boundary-value problem can be converted into an eigenvalue problem. Such an approach is efficient in handling the grating with an arbitrary profile and a finite stack of multiple gratings, as well. However, the RCWA method^1^ is known to be slowly converging for 1D metallic gratings in TM polarization (magnetic-field vector parallel to the grating vector). Fortunately, the inverse rule, by invoking adequate Fourier series of the permittivity and reciprocal permittivity functions of a periodic medium to reformulate the eigenvalue problem, was developed to achieved a highly improved convergence rate for the scattering analysis of a metallic grating in TM polarization^15–18^. Additionally, the Floquet modes in a periodic medium with the unit cell composed of a dielectric slab and a metal layer having finite conductivity can be determined by solving the dispersion equation^19^. However, finding their complex roots is a difficult task. Consequently, use of Fourier series expansion to calculate Floquet modes in a periodic medium, in general, is the most reliable and effective approach in handling a diffraction grating problem.

**Figure 1:**
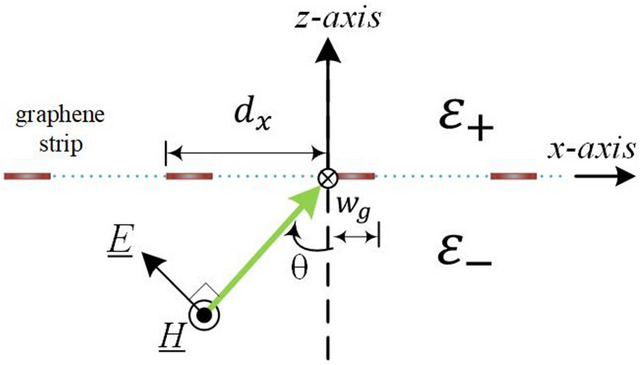
A periodic array of parallel graphene strips incident by a TM-polarized plane wave.

In this paper, we aimed at studying the numerical convergence of plane wave scattering by a graphene grating in TM polarization. Here, the structure under study is a 1D periodic array of graphene strips (ribbons) deposited on a dielectric substrate. The graphene sheet is assumed to be near-zero thickness (the thickness of mono-layer graphene is 0.335*nm*); therefore, the electromagnetic fields are merely in the upper and lower homogeneous mediums, as shown in Fig. 1. Due to the periodicity along the *x*-axis, electric and magnetic fields can be presented in the standard form of Floquet-Fourier series (or Rayleigh expansions). Moreover, the electrical property of graphene strips can be modeled with a surface conductivity ($\sigma _g$). Consequently, the graphene conductivity function in a unit cell is $\sigma (x)=\sigma _g$ on graphene strip and $\sigma (x)=0$ otherwise, which can be further expressed as a Fourier series. Furthermore, two electromagnetic boundary conditions including (1) the continuous of tangential electric fields across the graphene grating, and (2) the discontinuity of tangential magnetic fields across the graphene grating caused by the conduction current induced on graphene strips should be applied at the interface between two adjacent homogeneous mediums. Alternatively, such a problem amounts to imposing a periodic boundary condition on the tangential electric- and magnetic-fields at an interface between two uniform mediums. Moreover, the periodic boundary condition is obtained by expanding the graphene conductivity into a Fourier series expansion. The Laurent’s rule^16^ then can be applied for Fourier factorization of the conduction current $\sigma (x)E_x(x,z=0)$. By matching the Fourier coefficient corresponding to the same harmonic, an infinite set of linear equations for the input-output relation of the diffraction-order amplitudes are determined^5,7,20^. Notably, such an approach is termed as Fourier modal method (FMM) throughout this paper.

**Figure 2:**
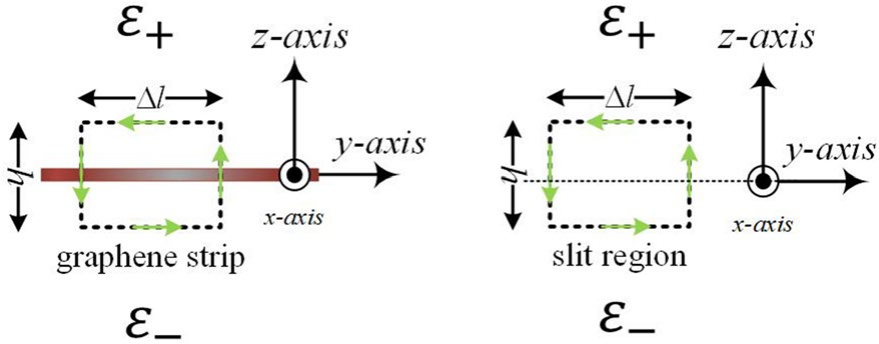
Derivation of approximate boundary condition in the graphene and slit regions.

Unfortunately, the poor convergence occurs in conventional FMM for the scattering analysis of periodic arrays of graphene ribbons reported in the literature^13,20^. Furthermore, the inverse rule by including the reciprocal function $1/\sigma (x)$ into the FMM is not applicable because $\sigma (x)$ is zero outside the graphene ribbon. To resolve this problem, the author^20^ proposed an approximate boundary condition (ABC) that takes into account the effective conductivity due to the displacement current in slit region without graphene. The effective conductivity is non-zero everywhere; therefore, the inverse rule can be successfully applied. Although FMM with ABC convincingly achieves numerical convergence, the convergent value varies in accordance with the enclosed-loop height (*h*), shown in Fig. 2, used to model the effective conductivity in slit region. Unfortunately, it is difficult to give a general criterion for *h*.

In this research, the respective local basis functions (LBFs) taking into account the electric-field edge conditions over the graphene-strip and slit regions in a unit cell are developed to replace the global basis functions (Floquet–Fourier series in a homogeneous medium). As will become clear later on, the tangential electric-field expanded by the local basis functions exhibits the fidelity of discontinuous behaviour, which is not seen in the conventional FMM, enabling a fast convergence in the scattering analysis of graphen-strip gratings in TM polarization.

This paper is organized as follows. We begin with the mathematical formulation using the conventional FMM that is taken as a general framework for theoretical analysis. Moreover, ABC is employed to reformulate the FMM by invoking the inverse rule. Additionally, the present approach, namely, FMM incorporating LBFs, will be comprehensively elaborated. Finally, convergence behaviour will be examined for the three aforementioned methods. Specifically, electric field distribution on graphene grating surface will be demonstrated for various incident conditions.

## Structure configuration and surface conductivity model of graphene

Figure 1 shows structure configuration of a 1D graphene grating consisting of a periodic array of parallel graphene strips. The strip is infinite in extent along the *y*-axis and the electromagnetic fields have no variation along that direction. The strip width and period along the *x*-axis are denoted as $w_g$ and $d_x$, respectively. The graphene layer is assumed to be zero thickness and characterized by a surface conductivity. Additionally, the 1D graphene strip array is sandwiched by two semi-infinite homogeneous mediums designed as $\varepsilon _{+}$ for $z \ge 0$ and $\varepsilon _{-}$ for $z < 0$, respectively. A plane wave with magnetic field ${\underline{H}}(x,z)={\hat{y}}H_y$ is impinging on the array; its incident angle is designated as $\theta$.

Incidentally, graphene conductivity ($\sigma _g=\sigma _{intra}+\sigma _{inter}$), having a close-form expression for the condition $\mid \mu _c \mid$ $\gg$ $k_B T$, consists of both the intraband ($\sigma _{intra}$) and interband ($\sigma _{inter}$) terms^11^:

1 $$\begin{aligned} \sigma _{intra}(\omega )= & {} \frac{2ie^2 k_B T}{\pi \hslash ^2(\omega +i\gamma )} \ln \left[ 2\cosh (\mu _c/2k_B T)\right] , \text{ and } \end{aligned}$$

2 $$\begin{aligned} \sigma _{inter}= \frac{e^2}{4\hslash } \left \{\frac{1}{2}+\frac{1}{\pi } \arctan \frac{\hslash (\omega +i\gamma )-2\mu _c}{2k_B T} -\frac{i}{2\pi }\ln \frac{[\hslash (\omega +i\gamma )+2\mu _c]^2}{\hslash (\omega +i\gamma )-2\mu _c]^2+(2k_BT)^2} \right \} , \end{aligned}$$

where -*e* is the electron charge, $\hslash$ is the reduced Planck constant, $\gamma$ is a phenomenological carrier scattering rate, $\mu _c$ is the chemical potential, $k_B$ is Boltzmann’s constant, and *T* is the ambient temperature.

## Fourier modal method

In a homogeneous medium, the electric and magnetic field components of TM polarization in the presence of periodicity $d_x$ along the *x*-axis can be represented by Rayleigh expansions (or Floquet–Fourier series) in the form

3 $$\begin{aligned} H_y(x,z)= & {} \sum _{n=-\infty }^{n=+\infty }I_n(z)\psi _n(x), \end{aligned}$$

4 $$\begin{aligned} E_x(x,z)= & {} \sum _{n=-\infty }^{n=+\infty }V_n(z)\psi _n(x), \end{aligned}$$

with $\psi _n(x)$ is named as the $n$th space harmonic given below

5 $$\begin{aligned} \psi _n(x)=\frac{exp(-jk_{xn}x)}{\sqrt{d_x}}. \end{aligned}$$

Function $\psi _n(x)$ forms an orthonormal set on $x\in [0, d_x]$ satisfying

6 $$\begin{aligned} \int _0^{d_x}\psi _m^{\dagger }(x)\psi _n(x) dx=\delta _{mn}. \end{aligned}$$

Parameter $\delta _{mn}$ is the Kronecker delta function, and $k_{xn}=k_o\sqrt{\varepsilon _{-}}\sin {\theta }+n2\pi /d_x$. Functions $V_n(z)$ and $I_n(z)$, satisfying transmission line equation, are the voltage and current amplitudes of the $n^{th}$ space harmonic, respectively. Their general solutions can be written in the form^5^

7 $$\begin{aligned} V_n(z)= a_n\exp (-jk_{zn}z)+b_n\exp (+jk_{zn}z), \end{aligned}$$

8 $$\begin{aligned} I_n(z)= & {} Y_n \left[ a_n\exp (-jk_{zn}z)-b_n\exp (+jk_{zn}z)\right] , \end{aligned}$$

with the propagation constant along the *z*-axis $k_{zn}=\sqrt{k_o^2\varepsilon _s-k_{xn}^2}$ and wave admittance $Y_n=\omega \varepsilon _o\varepsilon _s/k_{zn}$ in an infinite medium with relative dielectric constant $\varepsilon _s$. Parameters $a_n$ and $b_n$ usually are termed as the amplitudes of the forward- and backward- propagating waves of the $n^{th}$ diffraction order, respectively.

Owing to the zero thickness approximation of the graphene grating, discontinuity in the tangential component of magnetic fields at the interface (graphene grating surface) between two uniform mediums equals to the conduction current induced on the graphene strips array, yielding

9 $$\begin{aligned} H_y(x,z=0^{-})-H_y(x,z=0^{+})=\sigma (x)E_x(x,z=0), \end{aligned}$$

where graphene conductivity $\sigma (x)$ is a periodic function that can be expressed as a Fourier series

10 $$\begin{aligned} \sigma (x)= \left\{ \begin{array}{rcl} \sigma _g &{} \text{ for } &{} x\in \text{ graphene } \\ 0 &{} \text{ for } &{} x\in \text{ slit } \end{array} \right. =\sum _{n}\sigma _n \exp (jn2\pi x/d_x). \end{aligned}$$

We first substitute magnetic field $H_y$ in Eq. (3) and conductivity function $\sigma (x)$ in Eq. (10) into Eq. (9) together with the electric field $E_x$ on the graphene grating surface approximated by the Floquet-Fourier expansion in Eq. (4). Using Laurent’s rule^16^ and equaling the same Fourier coefficient corresponding to the same harmonic on both sides, one obtains the system of linear equations

11 $$\begin{aligned} \sum _{n=-\infty }^{n=+\infty } \left[ I_n(0^-)-I_n(0^+)\right] =\sum _{n=-\infty }^{n=+\infty } Y_{g,mn}V_n(0), \end{aligned}$$

where index *m* is running from negative to positive infinity.

Equation (11) can be expressed in a compact matrix form, one obtains

12 $$\begin{aligned} {\underline{I}}(0^{-})-{\underline{I}}(0^{+})=[[Y_g]]{\underline{V}}(0), \end{aligned}$$

where $[[Y_g]]$ is the Toeplitz matrix with (*m*, *n*) entry $\sigma _{m-n}$ given in Eq. (10); the $n$th element in column vectors ${\underline{I}}(0^{\pm })$ and ${\underline{V}}(0)$ respectively are $I_n(0^\pm )$ and $V_n(0)$ .

The matrix $[[Y_g]]$ in Eq. (12) establishes a relationship between voltage and current waves across the grating layer; it is the so-called admittance matrix in microwave engineering^5^. Equation (12) is also regarded as the input-output relation among incident, reflected and transmitted diffraction-order amplitudes with respect to a graphene grating.

Moreover, due to continuous of $E_x$ at $z=0$, Eq. (4) arrives at $V_n(0^-)=V_n(0^+)$; its vector form can be written as follow:

13 $$\begin{aligned} {\underline{V}}(0^+)={\underline{V}}(0^-)={\underline{V}}(0) . \end{aligned}$$

Furthermore, substitution of ${\underline{I}}(0^+)=[[Y_+]]{\underline{V}}(0^+)$ (no downward waves in the upper medium) and Eq. (13) into Eq. (12), one obtains

14 $$\begin{aligned} {\underline{I}}(0^-)=[[Y_{in}]]{\underline{V}}(0^-), \end{aligned}$$

where the input admittance matrix looking into the interface in lower medium is defined as $[[Y_{in}]]=[[Y_g]]+[[Y_{+}]]$.

Here the incident and reflected wave vectors, defined at $z=0^-$ in the lower medium, are individually denoted as column vectors ${\underline{a}}$ and ${\underline{b}}$ whose elements are $a_n$ and $b_n$, respectively. Since the voltage and current wave vectors at $z=0^-$ can be written as ${\underline{V}}(0^-)={\underline{a}}+{\underline{b}}$ and ${\underline{I}}(0^-)=[[Y_{-}]]({\underline{a}}-{\underline{b}})$ with their components given in Eqs. (7) and (8), the relationship between ${\underline{a}}$ and ${\underline{b}}$ can be obtained through Eq. (14). One obtains

15 $$\begin{aligned} {\underline{b}}=[[\Gamma ]]{\underline{a}} , \end{aligned}$$

where $[[\Gamma ]]$ is termed as the reflection matrix written below

16 $$\begin{aligned} {[}{[}\Gamma ]]=([[Y_-]]+[[Y_{in}]])^{-1}([[Y_-]]-[[Y_{in}]]) . \end{aligned}$$

Matrices $[[Y_{+}]]$ and $[[Y_{-}]]$ are denoted as the admittance matrices in the upper and lower uniform mediums, respectively; both are diagonal matrices with their $n^{th}$ diagonal entry given as

17 $$\begin{aligned} Y_n^{\pm }=\frac{\omega \varepsilon _o\varepsilon _{\pm }}{k_o \sqrt{\varepsilon _{\pm }-\varepsilon _{-}\sin ^2\theta }}. \end{aligned}$$

Since only the transmitted (upward) waves are present in the upper medium, the voltage vector in the upper medium at $z=0^+$ is written as ${\underline{V}}(0^+)={\underline{c}}$. By ${\underline{V}}(0^-)={\underline{a}}+{\underline{b}}$ together with Eq. (13), one obtains

18 $$\begin{aligned} {\underline{c}}=\left( [I]]+[[\Gamma ]\right) {\underline{a}} = [[T]]{\underline{a}} , \end{aligned}$$

where ${\underline{c}}$ is a column vector with element $c_n$ at the $n^{th}$ entry. Symbol [[*T*]] is termed as a transmission matrix defined at the output surface of grating ($z=0^+$); [[*I*]] is the identity matrix.

Notably, for a graphene grating incident by a single plane wave, the wave vector ${\underline{a}}$ is known and usually defined as $a_0=1$ and $a_n=0$ for $n\ne 0$. The amplitude of each reflected and transmitted diffraction order can then be obtained via Eqs. (15) and (18).

Moreover, the time average Poynting power along the *z*-axis over a grating period is defined as

19 $$\begin{aligned} P_z(z)=\frac{1}{2} {\text {Re}}\left[ \int _{0}^{d_x}E_x(x,z) H_y^{\dagger }(x,z)dx \right] =\frac{1}{2} {\text {Re}}\left[ \sum \limits _m\sum \limits _n V_m(z)I_n^{\dagger }(z)\int _{0}^{d_x}\psi _m(x)\psi _n^{\dagger }(x)dx\right] =\frac{1}{2} {\text {Re}}\left[ \sum _n V_n(z)I_n^{\dagger }(z)\right] . \end{aligned}$$

Therefore, the incident-, reflected- and transmitted- power are obtained as follows: $P_{inc}=\frac{1}{2}Y^{-}_0$, $P_{ref}=\frac{1}{2} {\text {Re}}\left[ \sum \limits _n Y_n^- |b_n|^2\right]$, and $P_{tx}=\frac{1}{2} {\text {Re}}\left[ \sum \limits _n Y_n^+ |c_n|^2\right]$, respectively. The absorptance can be obtained by evaluating $e_{abs}=1-P_{ref}/P_{inc}-P_{tx}/P_{inc}$.

Notably, the mathematical formulation in FMM is rigorous and the result is exact when the space harmonic index *n* runs from − $\infty$ to + $\infty$; however, they have to be truncate into $[-N,+N]$ in numerical computation, where *N* is denoted as truncated order. The total number of space harmonics is then designated as $N_{tot}$ equal to $2N+1$.

## Fourier modal method with approximate boundary condition

As reported in the literature^15, 16^, the slow convergence in the RCWA for TM polarization is not caused by the Fourier series expansion but the form where the Fourier series of the permittivity and the reciprocal permittivity functions are utilized. The same problem also occurs in graphene gratings with thickness of almost zero. Nevertheless, the inverse rule^15, 16^ is not applicable in the graphene grating because $1/\sigma (x)$ goes to infinity in the slit region. The approximate boundary condition on periodic arrays of graphene ribbons was proposed to replace $\sigma (x)$ by the effective conductivity function: $\sigma _{eff}(x)$ incorporating the contribution of displacement current^20^ in slit region. In doing so, the reciprocal function $1/\sigma _{eff}(x)$ exists everywhere. More precisely, by Ampere’s law with Maxwell’s modification, the discontinuity in magnetic fields intensity (*H*) on the graphene grating between two uniform mediums equals to the sum of conduction- and displacement-current. At the left-hand side of Fig. 2, the line integral over the graphene strip equals to the conduction current flowing along the *x*-axis; while it equals to the displacement current filled in the rectangular box at the right-hand side figure. Combining these two results, Eq. (9) can be rewritten as^20^

20 $$\begin{aligned} H_y(x,z=0^{-})-H_y(x,z=0^{+})=\sigma _{eff}(x)E_x(x,z=0), \end{aligned}$$

where the effective conductivity at $z=0$ is

21 $$\begin{aligned} \sigma _{eff}(x)=\sigma (x)+j\omega {\bar{\varepsilon }} h . \end{aligned}$$

Parameter $\sigma (x)$ is the graphen conductivity function given in Eq. (10) and ${\bar{\varepsilon }}=(\varepsilon _{+}+\varepsilon _{-})/2$. Parameter *h* is the height of the rectangular enclosed loop in Fig. 2.

Now, the reciprocal function $1/\sigma _{eff}(x)$ exists everywhere as well as its Fourier expansion. By the standard procedure of FMM in the previous section incorporating inverse rule^20^, we obtain the same input-output relation in Eq. (12) but with different admittance matrix, $[[Y_g]]=[[Z_g]]^{-1}$, where the (*m*, *n*) entry of $[[Z_g]]$ is given below:

22 $$\begin{aligned} z^{(g)}_{mn}=\int _{x=0}^{x=d_x}\frac{1}{\sigma _{eff}(x)}\exp [\frac{j(m-n)2\pi }{d_x}x] dx. \end{aligned}$$

Once the admittance matrix is obtained, the standard procedure of FMM in the previous section can be applied to calculate the reflection, transmission and absorption efficiencies.

## Fourier modal method incorporating local basis functions

Although FMM with ABC can improve the convergence rate, the parameter *h* is still a core factor affecting the value of convergence, as will be demonstrated in the next section. Moreover, the criterion for determining the appropriate *h* remains to be studied in detail. So far, we have implemented a computer program based on FMM with ABC for calculating the scattering characteristics of a graphene grating as well as the electric-field ($E_x$) distribution on the graphene grating surface. Let us skip ahead to the numerical result concerning a graphene-strip grating normally incident by a TM-polarized plane wave. Figure 5 shows the distribution of $E_x$ (red dotted curve obtained using FMM with ABC) over the graphene-strip ($x\in [0,20]$) and slit ($x\in [20,70]$) regions. It is obvious to see jump discontinuities occurring around $x=0$ and $x=20\mu m$; wherein $E_x$ (or $J_x/\sigma _g$) vanishes close to strip edges. In fact, induced current vanishes at strip edges can be explained physically as follows.

Referring to Fig. 1, at $x=w_g^+$ in the slit region, magnetic field component $H_y$ must be continuous across the interface due to $J_x(x=w_g^+,z=0)=0$, namely, $H_y(x=w_g^+,z=0^-)=H_y(x=w_g^+,z=0^+)$. Moreover, because of $H_y(x=w_g^+,z=0^-)=H_y(x=w_g^-,z=0^-)$ in the lower medium and $H_y(x=w_g^+,z=0^+)=H_y(x=w_g^-,z=0^+)$ in the upper medium, we have $H_y(x=w_g^-,z=0^-)=H_y(x=w_g^-,z=0^+)$, resulting in the vanished induced current density $J_x(x=w_g^-,z=0)$ obtained via Eq. (9); therefore we have $E_x(x=w_g^-,z=0)=0$, that is, vanishing $E_x$ at the edge. Additionally, the vanishing current at metal-strip edges was reported in the research of a metal-strip grating illuminated by a plane wave in TM polarization^19^, but unfortunately the distribution of $E_x$ in the slit region was not shown in that paper. Regarding the electric field in the slit region, an exponential growth of $E_x$ around the slit edges can be observed (red dotted curve) in Fig. 5. Alternatively, as is well know, TM-polarized electric field near the edge of a thin sheet is proportional to $\rho ^{-1/2}$ and becomes singular as $\rho$ approaches zero^21^, where $\rho$ is defined as the radius (in the polar coordinate system) with its original point locating at the edge.

Nevertheless, the Gibbs phenomenon taking place near the discontinuities reveals that the customary global basis of Floquet-Fourier series is inappropriate for expanding the field directly on the graphene grating surface. In view of that, it is essential to construct the local basis functions inherently satisfying the field nature in respective regions. Furthermore, the criterion for choosing basis functions contains: (1) to use only a few basis functions to approach the correct solution, and (2) to have closed forms in the overlap integral between the local basis functions and the space harmonic in Eq. (5).

More specifically, the $\sin$-based local basis function vanishing at its both ends for any harmonic order is used to expand $E_x$ over the graphene strip, which is given as

23 $$\begin{aligned} g_n(x)=\sqrt{\frac{2}{w_g}}\sin \frac{n\pi (x-x^{(g)}_1)}{w_g}, \end{aligned}$$

where $w_g=x^{(g)}_2-x^{(g)}_1$; the graphene strip belongs to the region of $[x^{(g)}_1, x^{(g)}_2]$; index *n* is ranging from 1 to $N_g$.

On the other hand, in the slit region, we have the singular basis functions with singularities at its two edges, which are commonly used to approximate the current parallel to the edges in a micro-strip line^22^. They are expressed as follows:

24 $$\begin{aligned} s_n(x)=\sqrt{\frac{\gamma _n}{w_s}}\frac{\cos \frac{n\pi (x-x^{(s)}_1)}{w_s}}{\sqrt{(w_s/2)^2-(x-x^{(s)}_c)^2}}, \end{aligned}$$

where $x^{(s)}_c=(x^{(s)}_1+x^{(s)}_2)/2$ and $w_s=x^{(s)}_2-x^{(s)}_1$; the slit is in the region of $[x^{(s)}_1, x^{(s)}_2]$. Parameter $\gamma _n=2$ for $n=0$ and $\gamma _n=1$ for $n\ne 0$; index *n* runs from 0 to $N_s-1$. Notably, the denominator in $s_n(x)$ approximates $\sqrt{w_s}\sqrt{\rho }$ at the strip edge where $x= w_g+\rho$ ($x_1^{(s)}=w_g$ and $x_2^{(s)}=d_x$), which confirms the electric-field edge condition described previously. Notably, Eqs. (23) and (24) both are expressed in the general form for easy extension to the case with multiple graphene strips in a period.

In a unit cell on the graphene grating surface (at $z=0$), $E_x(x)$ can be written as

25 $$\begin{aligned} E_x(x)= \left\{ \begin{array}{rcl} \sum \limits _{n=1}^{N_g} p_n g_n(x) &{} \text{ for } &{} x\in \text{ graphene } \\ \sum \limits _{n=0}^{N_s-1} q_n s_n(x) &{} \text{ for } &{} x\in \text{ slit } \end{array}. \right. \end{aligned}$$

Parameters $N_g$ and $N_s$ represent the number of basis in the graphene strip and slit regions, respectively.

Due to electromagnetic boundary condition, $E_x$ must be continuous at the interface between graphene grating and uniform medium at $z=0$. Therefore, equality of Eqs. (4) and (25) gives

26 $$\begin{aligned} \sum _{n=-N}^{n=+N}V_n(0)\psi _n(x)=\left\{ \begin{array}{rcl} \sum \limits _{n=1}^{N_g} p_n g_n(x) &{} \text{ for } &{} x\in \text{ graphene } \\ \sum \limits _{n=0}^{N_s-1} q_n s_n(x) &{} \text{ for } &{} x\in \text{ slit } \end{array}\right. . \end{aligned}$$

By multiplying the complex conjugate of $\psi _m(x)$ on both sides of Eq. (26) and taking integration over one period, we obtain

27 $$\begin{aligned} \sum _{n=-N}^{n=+N}V_n(0)\langle \psi _m(x)^\dagger | \psi _n(x)\rangle =\sum _{n=1}^{N_g}p_n \langle \psi _m(x)^\dagger | g_n(x)\rangle +\sum _{n=0}^{N_s-1} q_n \langle \psi _m(x)^\dagger | s_n(x)\rangle , \end{aligned}$$

where integer *m* is ranging from -*N* to +*N*. The notation of $\langle a(x) | b(x) \rangle =\int _{0}^{d_x}a(x)b(x) dx$ is defined as the overlap integral of functions *a*(*x*) and *b*(*x*) in the range of $[0,d_x]$. By the orthogonality of $\psi _n(x)$ in Eq. (6) and the closed form solutions of overlap integral, the above equation becomes

28 $$\begin{aligned} V_m(0)=\sum _{n=1}^{N_g}p_n G_{mn}+\sum _{n=0}^{N_s-1}q_n S_{mn}, \end{aligned}$$

with

29 $$\begin{aligned} G_{mn}= & {} \langle \psi _m(x)^\dagger | g_n(x)\rangle = \frac{-j}{\sqrt{2}}\sqrt{\frac{w_g}{d_x}} e^{jk_{xm} x^{(g)}_c} \cdot \left[ e^{jn\pi /2} sinc(\alpha _{mn}^{+} w_g/2)+ e^{-jn\pi /2} sinc(\alpha _{mn}^{-} w_g/2) \right] , \end{aligned}$$

30 $$\begin{aligned} S_{mn}= & {} \langle \psi _m(x)^\dagger | s_n(x)\rangle = \frac{\pi }{2}\sqrt{\frac{\gamma _n}{w_s d_x}}e^{jk_{xm}x^{(s)}_c} \cdot \left[ e^{jn\pi /2} J_o(\beta _{mn}^{+}w_s/2)+ e^{-jn\pi /2} J_o(\beta _{mn}^{-} w_s/2) \right] , \end{aligned}$$

where $\beta _{mn}^{\pm }=k_{xm}\pm n\pi /w_s$ and $\alpha _{mn}^{\pm }=k_{xm} \pm n\pi /w_g$; function $J_o(\cdot )$ is the zero order Bessel function of the first kind; function *sinc*(*x*) is the unnormalized sinc function defined as $sinc(x)=sin(x)/x$. Parameter *n* is the index of the local basis function.

Equation 28 can be rewritten as a matrix-vector form:

31 $$\begin{aligned} {\underline{V}}(0) = [[G]]{\underline{p}}+[[S]]{\underline{q}}= \begin{bmatrix} {[}[G]]&[[S]] \end{bmatrix} \begin{bmatrix} {\underline{p}} \\ {\underline{q}} \end{bmatrix} . \end{aligned}$$

Vector ${\underline{p}}$ is a $N_g$-by-1 column vector with its $n{\text{th}}$ element $p_n$; $q_n$ is the $n{\text{th}}$ element in column vector ${\underline{q}}$ of size $N_s$-by-1. Here, the sub-matrix [[*G*]] and [[*S*]] have the size $N_{tot}$-by-$N_g$ and $N_{tot}$-by-$N_s$, respectively. Specifically, $N_{tot}$, $N_g$ and $N_s$ satisfy the relationship: $N_{tot}=N_g+N_s$. Moreover, the ratio between $N_g$ and $N_s$ equals to the ratio of $w_g$ to $w_s$^5, 19^, therefore, we have $N_g=round[N_{tot}\cdot w_g/(w_g+w_s)]$ and $N_s=N_{tot}-N_g$; the operator *round*[.] rounds a real number towards the nearest integer. In doing so, we have a square matrix $\left[ [G]] \ [[S] \right]$ of size $N_{tot}$-by-$N_{tot}$.

Substituting of Eqs. (3) and (25) into Eq. (9), we obtain

32 $$\begin{aligned} \sum _{n=-N}^{n=+N}[I_n(0^-)-I_n(0^+)]\psi _n(x) =\sum _{n=1}^{N_g} \sigma _g p_n g_n(x). \end{aligned}$$

Multiplying $\psi _m^\dagger (x)$ on both sides of Eq. (32) and taking the integration over one period along the *x*-axis, one obtains

33 $$\begin{aligned} \sum _{n=-N}^{n=+N}[I_n(0^-)-I_n(0^+)]\langle \psi _m(x)^\dagger | \psi _n(x)\rangle =\sum _{n=1}^{N_g} \sigma _g p_n \langle \psi _m(x)^\dagger | g_n(x)\rangle . \end{aligned}$$

By invoking orthogonality and Eq. (29), the system of linear equations in Eq. (33) can be expressed in terms of matrix-vector form. One obtains

34 $$\begin{aligned} {\underline{I}}(0^{-})-{\underline{I}}(0^{+})= \begin{bmatrix} \sigma _g[[G]]&[[0]] \end{bmatrix} \begin{bmatrix} {\underline{p}} \\ {\underline{q}} \end{bmatrix} , \end{aligned}$$

where [[0]] is a null matrix of size $N_{tot}$-by-$N_s$, and matrix $\left[ \sigma _g[[G]] \ [[0]]\right]$ is a square matrix of size $N_{tot}$-by-$N_{tot}$.

After performing matrix operations with Eqs. (31) and (34), we obtain the Eq. (12) with a new admittance matrix of size $N_{tot}$-by-$N_{tot}$ given below

35 $$\begin{aligned} {[}[Y_g]]= \begin{bmatrix} \sigma _g[[G]]&[[0]] \end{bmatrix} \begin{bmatrix} {[}[G]]&[[S]] \end{bmatrix} ^{-1}, \end{aligned}$$

where the new admittance matrix in Eq. (35) is a square matrix of size $N_{tot}$-by-$N_{tot}$.

Here, we obtain a totally different admittance matrix while the input-output relation in Eq. (12) remains the same. The same procedure in FMM can be applied to calculate the reflect and transmit amplitudes of each diffraction order. Once the voltage ${\underline{V}}(0)=[[T]]{\underline{a}}$ is obtained, the expansion coefficients of local basis functions, ${\underline{p}}$ and ${\underline{q}}$ in Eq. (31) can be readily determined, as well as the distribution of $E_x$ on the graphene strip and slit.

Furthermore, the power dissipated on the graphene strips array can be directly determined by

36 $$\begin{aligned} P_{abs.}=\frac{1}{2}{\text {Re}}\left[ \sum _{n=1}^{n=N_g} \sigma _g \mid p_n \mid ^2 \right] . \end{aligned}$$

## Numerical results and discussion

A free-standing graphene-strip grating is taken as an example to examine the convergence behaviour for the three approaches. The parameters of graphene are $\mu _c=0.39\,{\text{eV}}$, $T=300\,{\text{K}}$, and $\hslash \gamma =0.658\,{\text{meV}}$ (relaxation time of charge carriers $\tau =1/2\gamma =0.5\,{\text{ps}}$). The period and strip width are $d_x=70\,\upmu {\text{m}}$ and $w_g=20\,\upmu {\text{m}}$, respectively. The upper and lower semi-infinite mediums are free space with $\varepsilon _{+}=\varepsilon _{-}=\varepsilon _o$.

**Figure 3:**
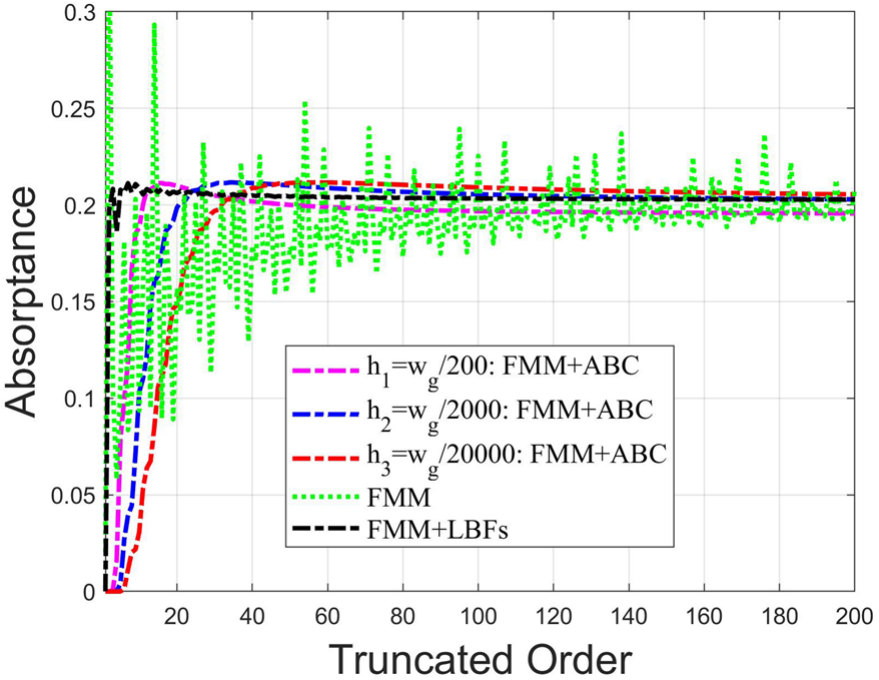
Absorptance against the truncated order *N*. The operating frequency and incident angle are 2.5 THz and $\theta =60{^{\circ}}$, respectively. The dotted curve in green colour is obtained by conventional FMM; the dashed curve in black colour is calculated by this approach (FMM incorporating LBFs); the dashed curve in red, blue and magenta colours individually correspond to $h=w_g/20000$, $h=w_g/2000$, and $h=w_g/200$, respectively, for the FMM with ABC^20^.

We first calculate the absorptance against the truncated order *N* running from 1 to 200. In Fig. 3, the three methods, including the conventional FMM, FMM with ABC, and the present approach FMM incorporating LBFs, were employed to carry out the convergence test. Three different enclosed-loop heights (*h*) in FMM with ABC are considered: $w_g/200$ (magenta dashed curve), $w_g/2000$ (blue dashed curve) and $w_g/20000$ (red dashed curve). As was widely reported in literature^15–18,20^, the conventional FMM (green dotted curve) indeed runs into the serious problem of oscillating convergence behaviour. On the other hand, the FMM with ABC can improve the convergence rate; however, the convergent value changes with *h* accordingly. Interestingly, the result with $h=w_g/2000$ approaches to that of our method. Apparently, the convergence rate of our approach (black dashed curve) is superior to the other two methods; even only a few number of truncated order is needed to achieve the numerical convergence.

**Figure 4:**
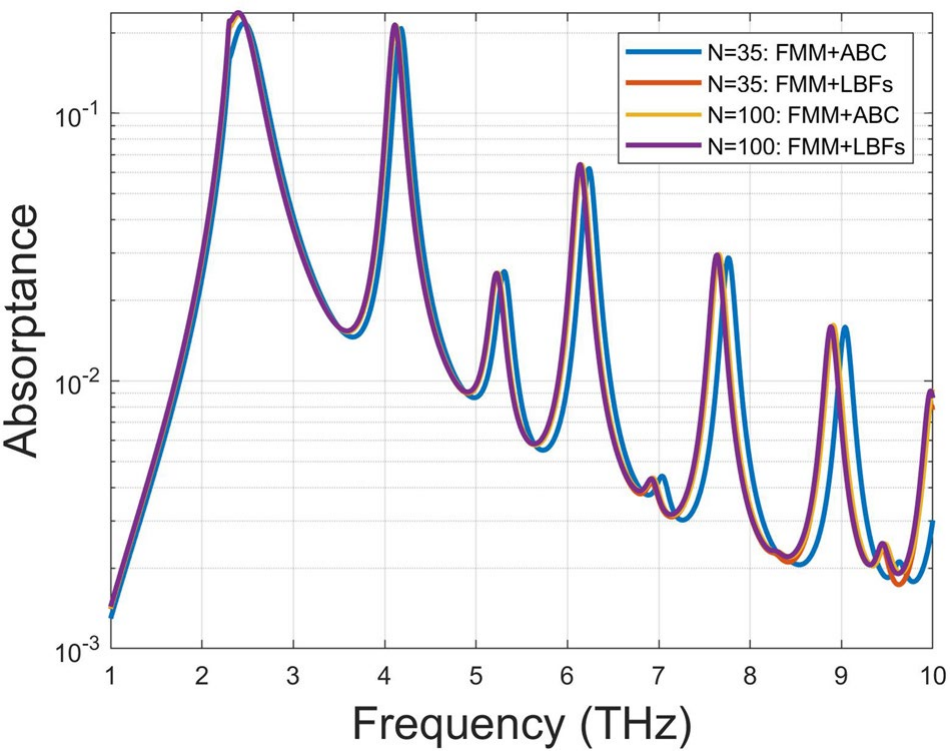
Absorptance versus frequency calculated by this approach and FMM with ABC for $h=w_g/2000$^20^; the incident angle is 60°; the number of truncated order is denoted as *N*. The curves in blue and yellow colours correspond to the results obtained by FMM with ABC of $N=35$ and $N=100$, respectively. The red and purple colours are for the cases of $N=35$ and $N=100$ based on our approach FMM incorporating LBFs.

Additionally, the absorption versus frequency for both convincing methods including the FMM with ABC and our approach are demonstrated in Fig. 4. Because of oscillating convergence in the conventional FMM, its result is unreliable and was neglected here. The aforementioned graphene and structure parameters are used in this example. The graphene grating is obliquely incident by a TM-polarized wave with incident angle 60°. The enclosed-loop height ($h=w_g/2000$) is chosen since it shares approximately the same convergence value with that of our approach in Fig. 3. Two truncated orders (*N*) are used to examine the performance of numerical convergence. It is obvious to see that FMM with ABC (yellow solid curve) and our approach (purple solid curve) agree very well for the case of $N=100$. Specifically, the result of our approach with $N=35$ (red solid curve) coincides with those of $N=100$. However, the result obtained by FMM with ABC for $N=35$ (blue solid curve) shows apparent discrepancy, particularly in the higher frequency range. Additionally, the Wood’s anomalous taking place at 2.3 THz can be observed in both approaches. We may conclude that compared to FMM with ABC, our approach can achieve numerical convergence even if only a few truncated orders is used.

**Figure 5:**
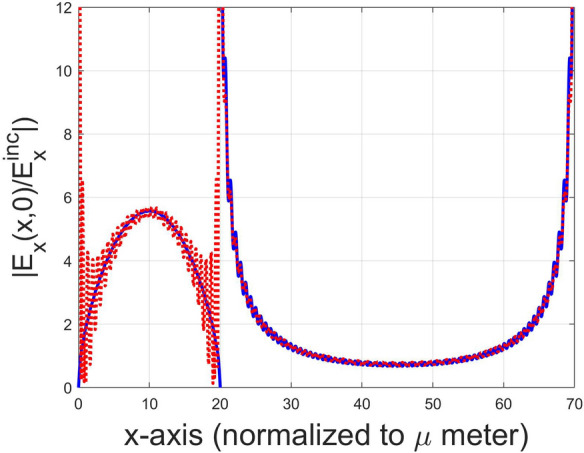
Comparison of $|E_x(x,z=0)|$, normalized to the incident $E_x$ in a unit cell on the graphene grating surface using the FMM with ABC (dotted curve in red colour) and our approach incorporating LBFs in FMM (solid curve in blue colour). The operating frequency and incident angle are 2.5 THz and $\theta =0{^{\circ}}$, respectively. The truncated order is $N=100$. The region for $x\in [0,20\,\upmu m]$ is in the graphene strip; otherwise is in the slit region.

Figure 5 depicts the absolute value of $E_x(x,z=0)$, which was normalized to the incident $E_x$, versus *x* in a unit cell on the graphene grating surface. The red dotted curve is calculated based on FMM with ABC, while the solid curve in blue colour is obtained using FMM incorporating LBFs. The region for $x\in [0, 20\mu m]$ is the graphene strip, and is otherwise the slit region. Although the Gibbs phenomenon, an overshoot (oscillating) of a Fourier series occurring at jump discontinuities, is obvious in red dotted curve based on FMM with ABC, the vanishing current density ($\sigma _g E_x$) at the strip edges and exponentially growth in $E_x$ around the slit edges can still be clearly observed. Contrarily, owing to the two local basis functions, given in Eqs. (23) and (24), inherently satisfy the individual edge condition, the Gibbs phenomenon is removed, as shown in the blue solid curve. Although not shown here, the case of $N=35$ shares almost the same profile with the case of $N=100$ ; this means that only a few LBFs is needed to expand $E_x$. Notably, in this case the FMM with ABC can achieve almost the same result of absorptance for the case of $N=100$; however, it can not reflect the essence of field nature around the graphene strip edges. Additionally, the operating frequency 2.5 THz is near the absorption peak; the incident wave is resonant with the graphene current $J_x$ along the *x*-direction. Therefore, the induced current exhibits the first normal mode (standing wave) pattern. It is very similar to the current induced on a radio-frequency (RF) dipole antenna excited at its first resonant frequency.

**Figure 6:**
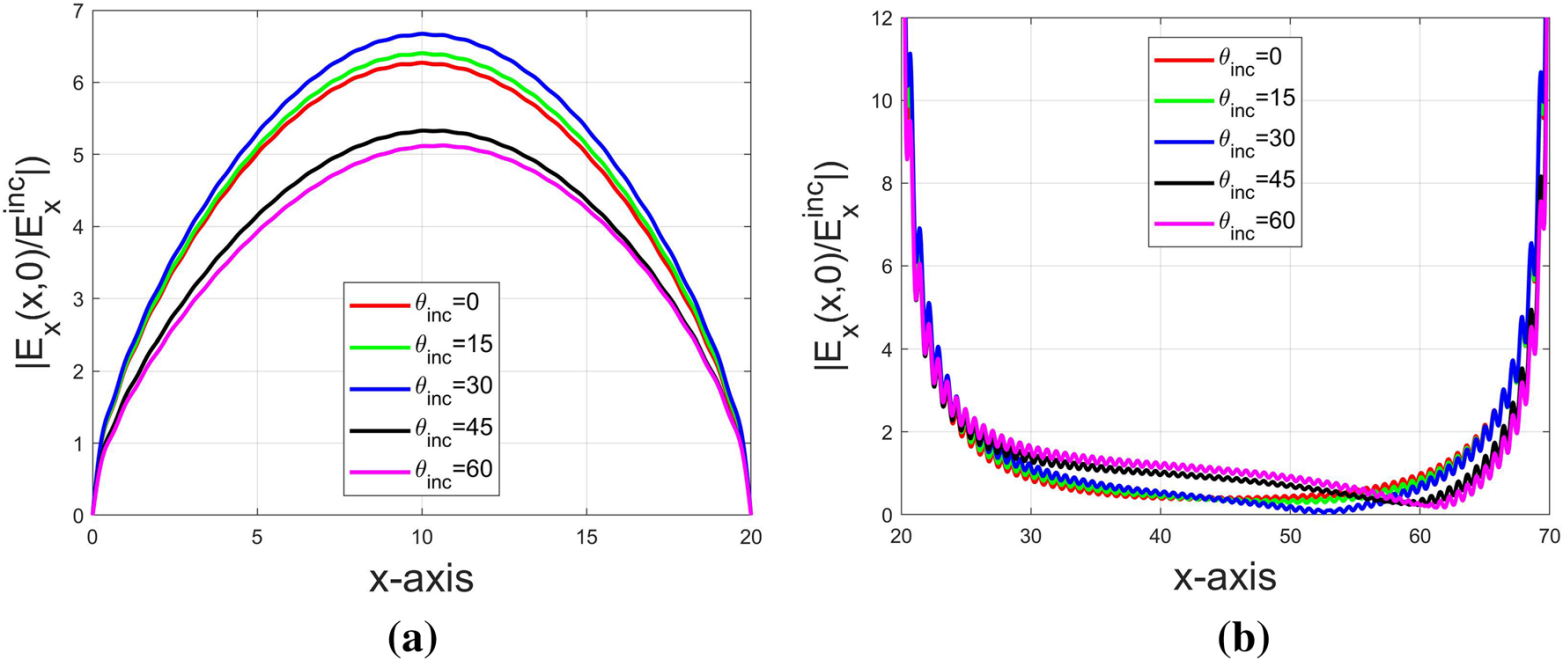
Distribution of normalized $E_x$ on the graphene grating surface in a unit cell for various incident angles with the operating frequency 2.6 THz: (**a**) $|E_x(x,z=0)/E^{inc}_x|$ on graphene strip, and (**b**) $|E_x(x,z=0)/E^{inc}_x|$ over slit.

Since the approach of FMM incorporating LBFs can effectively remove the Gibbs phenomenon, it should be in a good position to observe the effect of incident angles on the electric field ($E_x$) distribution over the graphene-strip grating surface. In Fig. 6a and b, the absolute value of $E_x(x,z=0)$ normalized to the incident $E_x$ on the graphene grating surface against *x*-axis was demonstrated for the five cases with different incident angles including 0°, 15°, 30°, 45°, and 60°, respectively. Fig. 6a shows the electric field strength over the graphene strip, while Fig. 6b shows that in the slit region at $z=0$. As shown in Fig. 6a, the induced $E_x$ (or $J_x/\sigma _g$) on the graphene strip changes insignificantly as $\theta$ is increasing from 0° to 30°, while its peak is decreasing as the incident angle increases up to 45° and 60°. Interestingly to find that the current distribution is almost symmetric with respect to the graphene strip centered at $x=10\,\upmu m$ even for the oblique incidence. As was reported in literature^13^, the resonance effects are in connection with the leaky plasmonic modes existing in individual graphene strip but with weak coupling between strips. Such a normal mode is a source-free solution; therefore, its mode pattern is almost independent of the incident angle. Contrarily, the asymmetric distribution is observed in the slit region for oblique incidence, which maybe caused by unbound waves with continuous spectrum.

## Conclusion

In this research, the three approaches including the conventional FMM, FMM with ABC, and our approach incorporating LBFs in FMM were implemented to examine the convergence behaviour of absorptance with respect to a periodic array of parallel graphene strips obliquely incident by a TM-polarized plane wave. Because of the individual local basis functions inherently satisfying the electric-field edge condition at graphene-strip and slit edges, the Gibbs phenomenon due to the Fourier expansion of global basis functions (space harmonic) in conventional FMM disappears. Furthermore, the convergence rate of the present approach is superior to the other two methods. Additionally, a new admittance matrix is obtained in the present approach while the whole formulation can still fit into the standard procedure of FMM. Significantly, the inverse rule and ABC for FMM are no longer needed. Such an approach can drastically reduce the required number of space harmonics and is more efficient for scattering analysis of stacked multiple graphene gratings.
